# OTME-1. TAMEP are brain tumor parenchymal cells controlling neoplastic angiogenesis and progression

**DOI:** 10.1093/noajnl/vdab070.052

**Published:** 2021-07-05

**Authors:** Roland Kälin, Linzhi Cai, Yuping Li, Louisa von Baumgarten, Christian Schulz, Ines Hellmann, Rainer Glass

**Affiliations:** 1 University Clinics Munich, Munich, Bavaria, Germany; 2 Ludwig Maximilians University, Munich, Munich, Bavaria, Germany

## Abstract

Aggressive brain tumors like glioblastoma depend on support by their local environment and subsets of tumor parenchymal cells may promote specific phases of disease progression. We investigated the glioblastoma microenvironment with transgenic lineage-tracing models, intravital imaging, single-cell transcriptomics, immunofluorescence analysis as well as histopathology and characterized a previously unacknowledged population of tumor-associated cells with a myeloid-like expression profile (TAMEP) that transiently appeared during glioblastoma growth. TAMEP of mice and humans were identified with specific markers. Notably, TAMEP did not derive from microglia or peripheral monocytes but were generated by a fraction of CNS-resident, SOX2-positive progenitors. Abrogation of this progenitor cell population, by conditional Sox2-knockout, drastically reduced glioblastoma vascularization and size. Hence, TAMEP emerge as a tumor parenchymal component with a strong impact on glioblastoma progression.

